# Stability of the knee after posterior cruciate ligament reconstruction using peroneus longus tendon graft with three femoral insertion sites. A cadaveric study

**DOI:** 10.1016/j.heliyon.2022.e11972

**Published:** 2022-12-05

**Authors:** Duong Binh Tran, Thi Cao

**Affiliations:** a Department of Orthopaedics, Cho Ray Hospital, Ho Chi Minh City, Viet Nam; b Department of Orthopaedics and Rehabilitation, Faculty of Medicine, University of Medicine and Pharmacy at Ho Chi Minh City, Ho Chi Minh City, Viet Nam

**Keywords:** Posterior cruciate ligament, Knee stability, Ligament reconstruction, Bundle, Peroneus longus tendon, PCL

## Abstract

**Introduction:**

Many kinds of grafts were used for single-bundle reconstruction of the posterior cruciate ligament (PCL). Recently, the peroneus longus tendon (PLT) was used in some clinical reports. This study aimed to test the best position of the femoral insertion in the case of using PLT for PCL reconstruction.

**Methods:**

Seventeen fresh frozen cadaveric knees were randomized into three groups. Group AL (6 knees): the femoral insertion in PCL reconstruction was at the footprint center of the anterolateral bundle (ALB). Group PM (5 knees): at the footprint center of the posteromedial bundle (PMB). And group MC (6 knees) was at the midpoint of the center of the anterolateral bundle and posteromedial bundle. The PCL of all knees was cut and a PCL reconstruction procedure was performed with autologous peroneus longus tendon (PLT). The stability of each knee was tested in three conditions: PCL was intact, PCL was resected, and PCL was reconstructed. The KT-1000 machine was used to measure the maximum posterior displacement of the tibia under force with the knees at 0, 30, 60, 90, and 120 degrees of flexion.

**Results:**

Average posterior displacement of the tibia under force for intact PCL of group AL was 1.6 mm, group MC was 1.2 mm, and group PM was 1.3 mm. After PCL was resected, the knee laxity was increased remarkably: posterior displacement of the tibia of group AL was 8.9 mm, group MC was 9.4 mm, and group PM was 13.6 mm. After PCL was reconstructed, group AL was 1.5 mm, group MC was 2.0 mm, and group PM was 5.6 mm. The results showed that after PCL reconstruction the group AL and group MC give better stability to the knee (p < 0.05, except knee at 120 degrees of flexion). Group AL got more stability than group MC, but the difference was not significant (p ≥ 0.164)

**Conclusion:**

In a single-bundle reconstruction of the PCL with the graft PLT, the femoral insertion at the footprint center of the ALB and the midpoint of the center of the ALB and PMB showed better stability than that at PMB.

## 1 Introduction

Despite advances in knowledge of the basic science and clinical practice of the posterior cruciate ligament (PCL), some issues still need further definition. It is recommended that PCL reconstruction should be performed in patients with more than grade II PCL injury, even for isolated PCL injury in young patients [1, 2]. While there were superior results of double-bundle reconstruction to single-bundle reconstruction in biomechanical studies, there is no clinical evidence showing the superiority of functional outcomes after double-bundle PCL reconstruction [3, 4]. Likewise, the expenditure on double-bundle reconstruction is much more than on single-bundle reconstruction. Therefore, many surgeons still perform single-bundle reconstruction.

The site of femoral insertion in single-bundle reconstruction can be at the footprint center of the anterolateral bundle (ALB), posteromedial bundle (PMB), or the midpoint of the center of ALB and PMB (MC). There were some studies on the influence of the femoral attachment sites in single-bundle reconstruction. It was concluded that the center of ALB might be the best site. In almost previous clinical and experimental studies, the grafts were often semitendinosus and gracilis tendons, bone-patellar tendon-bone, quadriceps tendon, or Achilles tendon. Recently, because harvesting PLT did not cause significant morbidity at the donor site [5, 6] and the tensile strength of the peroneus longus was comparable to the hamstring tendon and was significantly more potent than the patellar tendon and quadriceps tendon [7, 8], many authors used PLT as a graft for PCL reconstruction [9, 10]. It is hypothesized that PCL reconstruction using PLT graft with the femoral insertion site at ALB will give the best stability of the knee more than other sites. Therefore, an experimental study of that procedures must be performed. In this study, the posterior instability of cadaveric knees was tested in three conditions: intact, resected, and reconstructed PCL using PTL graft, to determine which site of femoral insertion will give the best stability to the knee.

## 2 Materials and methods

**Figure 1:**
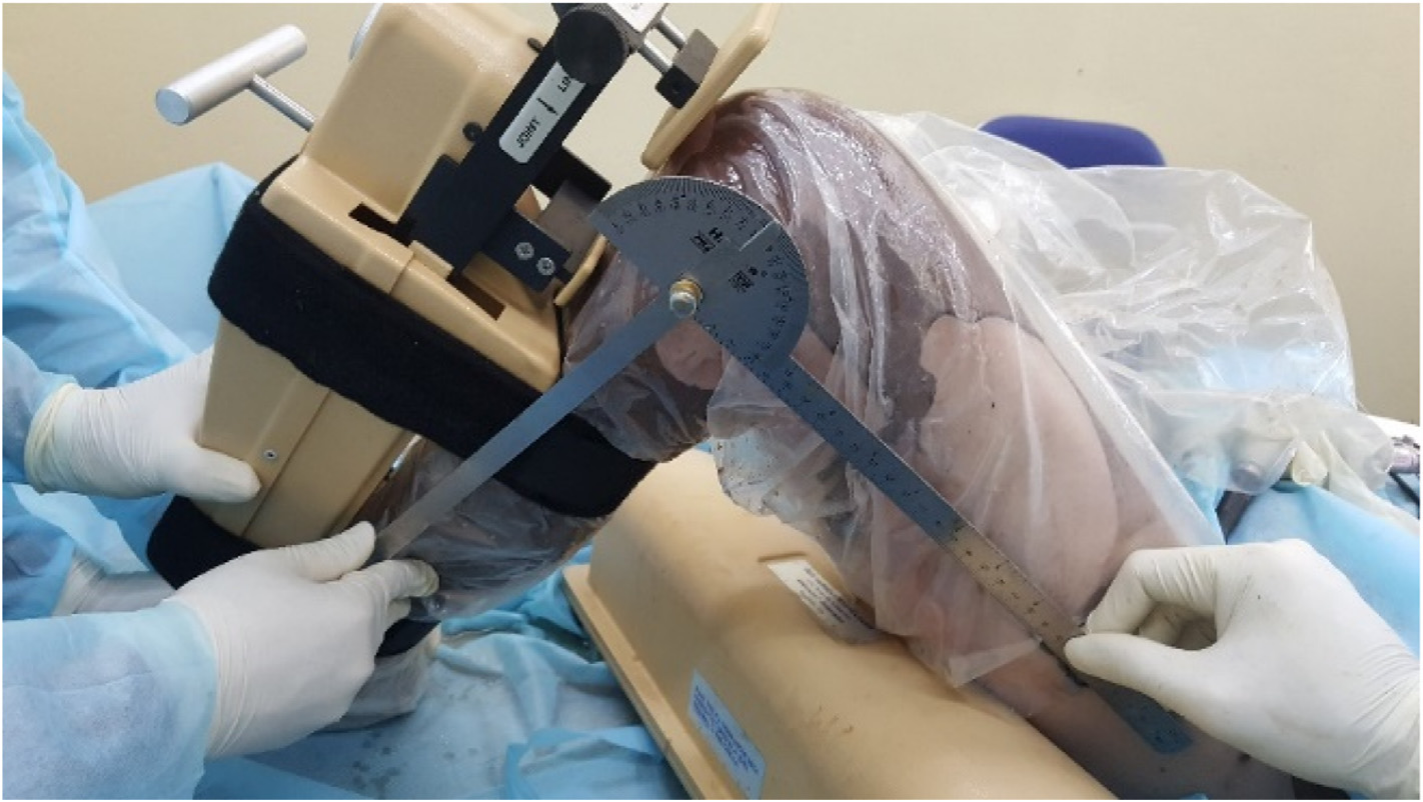
The posterior laxity of the knee was checked by the KT-1000 machine.

**Figure 2:**
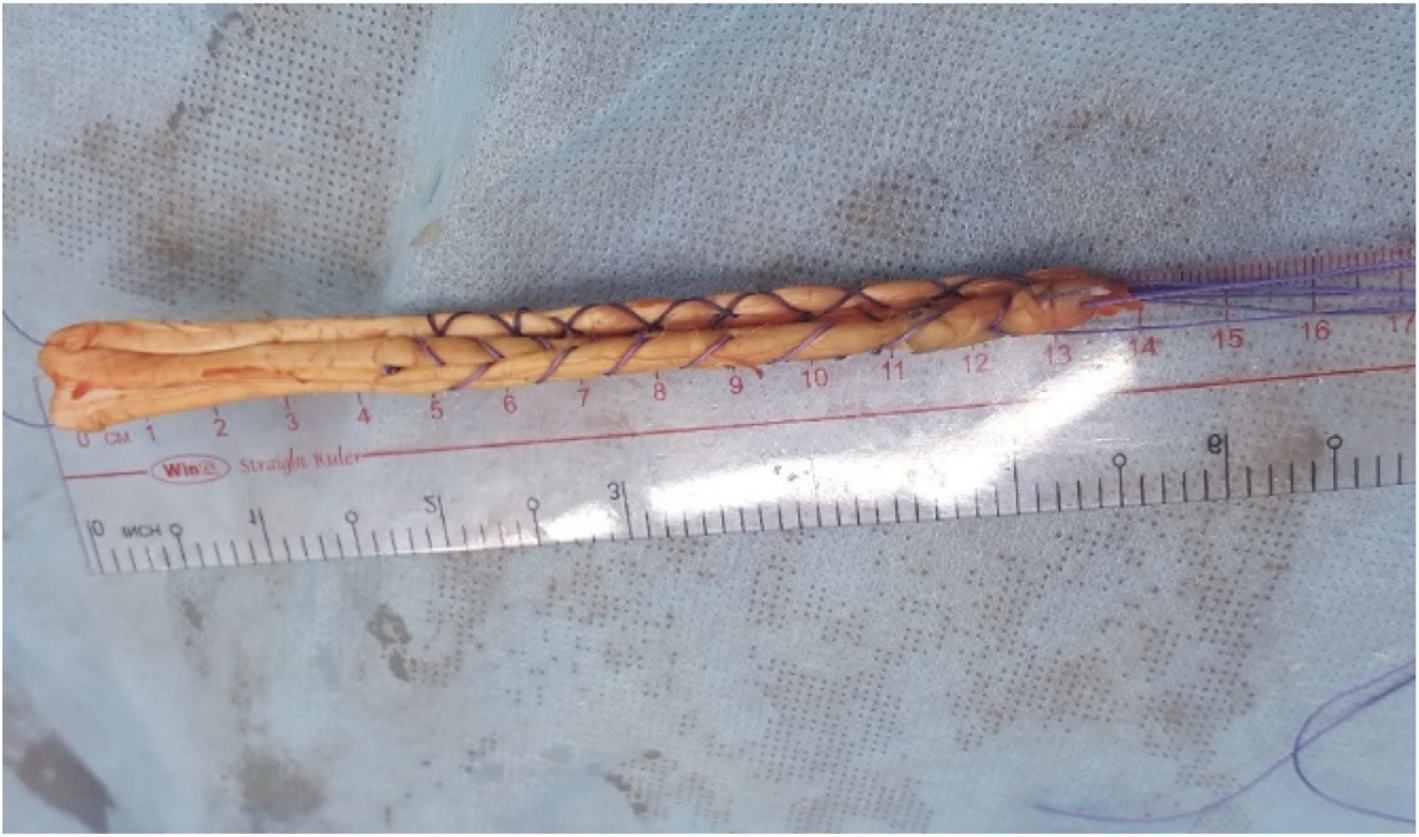
The processed peroneus longus tendon graft.

**Figure 3:**
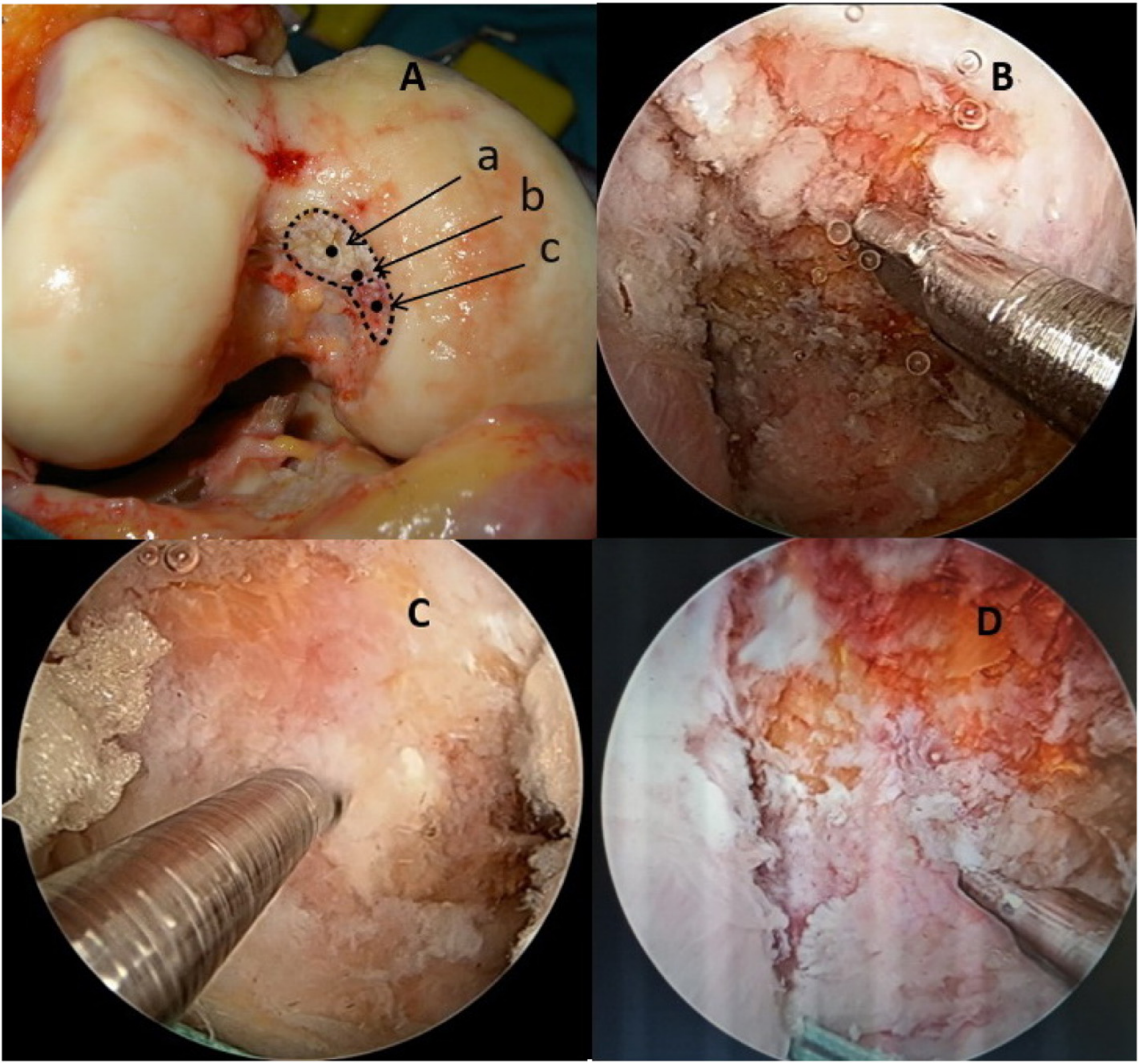
The attachment sites on the medial condyle for PCL reconstruction. A: Illustration for tunnel opens on medial condyle; a: center of the ALB footprint, b: midpoint of the center of ALB and PMB footprint, c: center of the PMB footprint B: At the center of the ALB footprint (group AL). C. At the midpoint of the center of ALB and PMB footprint (group MC). D. At the center of the PMB footprint (group PM).

Seventeen specimens, including seven pairs of knees and three single knees of 10 fresh frozen cadavers (7 males and three females) aged from 44 to 88 (average 72.6), were dissected, and data were recorded. The exclusion criteria consisted of any signs of lesions or tumors causing deformation of anatomy, structure, or congenital malformations of the thigh over the knee. After defrosting, the cadavers were put in a supine position like patients on the operating room table. The KT-1000 machine was used to check posterior laxity of the knee in 0°, 30°, 60°, 90°, and 120° flexion under a force of 89 N (Figure 1). An arthroscopy system was used to explore inside the knee and PCL was cut by saver or radiofrequency ablation. The posterior laxity was rechecked in the same position. Then, the ipsilateral peroneus longus tendon was harvested to use as a graft for reconstruction. The peroneus longus tendon was processed to become a graft suitable for reconstruction with femoral condyle wire-loop and tibial interference screw (Figure 2). Cadaveric knees were randomized into three groups. Group AL, in which the femoral attachment sites were located at the center of the ALB footprint, consisted of 6 knees. Group PM, in which the femoral attachment sites were situated in the center of the PMB footprint, consisted of 5 knees. Group MC, in which the femoral attachment sites were located at the midpoint of the center of ALB and PMB footprint, consisted of 6 knees (Figure 3).

The tibial attachment sites of all cases were at the center of the tibial footprint. After the graft was put in the tunnels and hung by a fixed-length loop, the graft was stretched in 90° flexions of the knee in the reconstruction procedure until the "step-off" sign disappeared. A screw 1 mm bigger than the pass-through caliber of the graft was used to fix the graft in the tibial tunnel. Then, the laxity of the knee was checked by the KT-1000 machine, in the same way, as before cutting PCL. An Excel file was used to record the obtained data and statistical tests such as the Kruskal-Wallis test, and Mann-Whitney test were used to analyze the data.

The research project was approved by the Bioethics Commission of the University of Medicine and Pharmacy at Ho Chi Minh City, Vietnam. The legal document related was Bioethics Commission Approval: 32/ĐHYD-HĐ on 30th January 2018. All written informed consents for the cadavers were obtained before they passed away.

## 3 Results

In all 17 specimens of the study, after processing, the average pass-through caliber of peroneus longus grafts was 8.2 mm (8–9 mm) (group AL = 8.1, MC = 8.3, PM = 8.3, p = 0.279/Kruskal-Wallis test), and the average length was 12.4 cm (11–14 cm). After the PCL was reconstructed, the average posterior laxities of groups AL, MC, and PM were 1.5 ± 0.8, 2.0 ± 1.5, and 5.6 ± 1.6 mm, respectively.

The posterior displacements of the tibia in the case of intact PCL, resected PCL, and reconstructed PCL were given in Tables 1, 2, and 3. Two more values were calculated. (1) The decrease of laxity of reconstructed PCLs compared with resected PCLs, equals the tibial posterior displacement after resection minus the tibial posterior displacement reconstruction was given in Table 2. (2) The increase of laxity of reconstructed PCLs compared with intact PCLs, equals the tibial posterior displacement reconstruction minus the initial tibial posterior displacement was given in Table 3.

**Table 1:** Posterior displacements of the tibia (mm) of AL group (n = 6), MC group (n = 6), and PM group (n = 5).

Knee flexion	Group	intact PCL	resected PCL	reconstructed PCL	*p*-value (Kruskal-Wallis test)
0°	AL	0.7 ± 0.4	5.2 ± 1.3	0.9 ± 0.6	0.002
	MC	1.0 ± 0.6	7.8 ± 2.6	1.1 ± 0.7	0.003
	PM	0.9 ± 0.2	11.5 ± 3.7	4.9 ± 0.5	0.002
30°	AL	2.4 ± 1.9	9.7 ± 2.5	1.6 ± 0.7	0.003
	MC	1.3 ± 0.6	9.9 ± 3.4	1.8 ± 1.3	0.003
	PM	1.3 ± 0.3	13.9 ± 1.2	5.8 ± 1.6	0.002
60°	AL	1.8 ± 0.5	9.4 ± 3.3	1.8 ± 0.7	0.003
	MC	1.2 ± 0.5	10.9 ± 2.3	1.8 ± 1.2	0.003
	PM	1.3 ± 0.3	13.9 ± 2.5	5.3 ± 0.8	0.002
90°	AL	1.8 ± 1.0	10.9 ± 3.8	1.5 ± 0.8	0.003
	MC	1.3 ± 0.6	9.2 ± 2.9	2.5 ± 1.5	0.002
	PM	1.6 ± 0.7	14.6 ± 2.4	6.5 ± 2.5	0.002
120°	AL	1.4 ± 0.9	9.4 ± 4.3	1.6 ± 1.0	0.003
	MC	1.1 ± 0.6	9.0 ± 2.8	3.0 ± 2.0	0.002
	PM	1.5 ± 0.4	14.0 ± 3.4	5.7 ± 2.0	0.002
Average of group AL		1.6 ± 1.1	8.9 ± 3.5	1.5 ± 0.8	
Average of group MC		1.2 ± 0.6	9.4 ± 2.8	2.0 ± 1.5	
Average of group PM		1.3 ± 0.4	13.6 ± 2.8	5.6 ± 1.6	
- Group AL: the femoral attachment sites were located at the center of the ALB footprint - Group MC: the femoral attachment sites were located at the midpoint of the center of ALB and PMB footprint - Group PM: the femoral attachment sites were located at the center of the PMB footprint

**Table 2:** The decrease of laxity of reconstructed PCLs compared with resected PCLs (mm).

Knee flexion	Group AL (n = 6)	Group MC (n = 6)	Group PM (n = 5)	*p*-value (Kruskal-Wallis test)
0°	4.3	6.7	6.6	p = 0.224
30°	8.1	8.1	8.1	p = 0.998
60°	7.6	9.1	8.6	p = 0.681
90°	9.4	6.7	8.1	p = 0.172
120°	7.8	6.0	8.3	p = 0.325
Average	7.4	7.7	7.9	
- Group AL: the femoral attachment sites were located at the center of the ALB footprint - Group MC: the femoral attachment sites were located at the midpoint of the center of ALB and PMB footprint - Group PM: the femoral attachment sites were located at the center of the PMB footprint

**Table 3:** The increase of laxity of reconstructed PCLs compared with intact PCLs (mm).

Knee flexion	Group AL (n = 6)	Group MC (n = 6)	Group PM (n = 5)	p-value (Kruskal-Wallis)	*p*-value (Mann-Whitney test)		
					AL versus MC	AL versus PM	PM versus MC
0°	0.3	0.1	4.0	p = 0.006	0.471	**0.006**	**0.004**
30°	-0.8	0.5	4.5	p = 0.004	0.164	**0.006**	**0.006**
60°	0.0	0.6	4.0	p = 0.008	0.459	**0.006**	**0.010**
90°	-0.3	1.2	4.9	p = 0.017	0.330	**0.010**	**0.028**
120°	0.2	1.9	4.2	p = 0.031	0.169	**0.013**	**0.118**
Average	-0.1	0.9	4.3				
- Group AL: the femoral attachment sites were located at the center of the ALB footprint - Group MC: the femoral attachment sites were located at the midpoint of the center of ALB and PMB footprint - Group PM: the femoral attachment sites were located at the center of the PMB footprint							

## 4 Discussion

The most important result of the present study was to define the best insertion site on the femoral condyle for the PCL reconstruction, in which the graft was autologous PLT. There were some studies on the tensile of the PLT, and the conclusion was that PLT qualified to be a graft for ACL or PCL reconstruction [7, 8]. In this study, only the dimensions of PLT were measured. As a result, the average diameter of the graft was 8.2 mm. This dimension was large enough to qualify for a PCL graft and this result was similar to those of some previous studies [9, 11, 12]. The graft calibers of the three groups AL, MC, and PM were not significantly different, so they did not affect the laxity of the knee after PCL reconstruction. Despite the length of the processed grafts being various, the shortest processed graft was 11 mm, enough for PCL reconstruction. It was thought that PLT samples of this study could be a relative representation.

Despite the knee being normal and the PCL intact, there was still a posterior displacement of the tibia under thrust. On the KT-1000 machine, the posterior displacements of the tibia were 0.7–2.4 mm, and they were the same in different knee flexions. At the five positions of the knee flexion of the three groups, the average posterior laxity of all specimens was 1.4 mm. This result was similar to that of Noyes. In Noyes' study, the posterior displacement of the tibia was 0–3 mm [13]. Lenschow studied ten defrosted cadavers in.

which Robot KuKa thrust the tibia with 134 N force, which had the posterior displacement of the tibia 3.7–6.4 mm [14], bigger than that of this study. The reason may be the force in this study was smaller than those of Lenschow's.

After the PCL was resected, the posterior laxity of the tibia increased remarkably. The average posterior laxity of all specimens at five positions of the knee flexion was 10.5 mm, and it got the biggest values at 60^0^-90° knee flexion. This result was similar to many authors [13, 14, 15].

After PCL was resected, the laxity increased, but the increase was different between the three groups. The increase (compared with the initial) for groups AL, MC, and PM was about 7.3, 8.2, and 12.3 mm, respectively. This big difference caused difficulty in evaluating the effectiveness of reconstruction. Therefore, in this study, besides the posterior laxity in any knee position of the three situations (initial, resected, and reconstructed PCL), two more values were calculated. The decrease in posterior laxity of reconstructed PCLs compared with resected PCLs showed the effectiveness of reconstruction. In Table 2, the decrease of posterior laxity of reconstructed PCLs compared with resected PCLs of groups AL, MC, and PM were similar at all flexion positions (p > 0.05). However, after PCL was resected, the laxity was different between the three groups. There must be a comparison between reconstructed PCLs and intact PCLs. Table 3 demonstrates another effectiveness of PCL reconstruction. It shows whether the procedure can regain the initial stability of the knee or not. On this view, in Table 3, group PM got the biggest laxity with a laxity bigger by at least 4.0mm than that of the intact PCL. This result was very different from those of the group's AL and MC. The posteromedial bundle footprint is not a good insertion site for PCL reconstruction. For the group AL and MC, group AL almost regained the normal laxity after reconstruction, while group MC had a laxity 0.9 mm bigger than that of the initial condition, but the differences are not significant (p > 0.05). At any flexion positions 30°, 60°, and 90°, the results were not remarkably different. Thus, as the results of this study, the ALB footprint is the best insertion site for PCL reconstruction, but the MC footprint is also the acceptable site.

There have been some studies using different grafts that gave the same results as this study. Pereira performed PCL reconstruction on cadaveric knees with femoral attachment at the center of the ALB, using a big graft, which gave posterior stability similar to the initial state. Moreover, the study also showed that the stability of the single bundle was similar to the double-bundle reconstruction [16]. Although some studies recognized a residue of posterior laxity after PCL reconstruction with the femoral insertion at the center of ALB [17, 18], the results of this present study showed that the knee could regain its initial stability. Lenschow [14] did PCL reconstruction on ten knees and concluded that placing a graft at the center of the femoral attachment does not restore normal posterior laxity. This present study had the same result, despite a bit of difference between MC and PM groups.

Like cadaveric biomechanical studies, this study has limitations. The sample size was small and almost cadavers were very old of age so the outcomes could be affected. In measurement, the dimensions are too small so that the result can be approximate. While comparing the result between groups, several interfering factors could not be controlled.

In conclusion, this study showed that the PCL reconstruction using PLT and the femoral insertion at the center of the ALB footprint and the midpoint of the ABL and PMB footprint could give better stability to the knee. The ABL footprint got more stability than the midpoint of the centers of ALB and PMB footprint but the difference was not significant. However, this study did not mention the graft kinematic, and clinical relevance needs more studies.

## Declarations

### Author contribution statement

Duong Tran Binh: Conceived and designed the experiments; Performed the experiments; Analyzed and interpreted the data; Contributed reagents, materials, analysis tools or data.

Thi Cao: Conceived and designed the experiments; Performed the experiments; Analyzed and interpreted the data; Contributed reagents, materials, analysis tools or data.

### Funding statement

This research did not receive any specific grant from funding agencies in the public, commercial, or not-for-profit sectors.

### Data availability statement

Data will be made available on request.

### Declaration of interest's statement

The authors declare no conflict of interest.

### Additional information

No additional information is available for this paper.
